# Coexistence of Triphasic Diabetes Insipidus and Cerebral Salt Wasting Syndrome Following Extraction of Sellar/Suprasellar Grade I Pilocytic Astrocytoma

**DOI:** 10.7759/cureus.17661

**Published:** 2021-09-02

**Authors:** Khalid Alghamdi, Lamair A Albakri, Yazeed Alotaibi, Ahmed H Alghamdi, Salwa Alaidarous

**Affiliations:** 1 Neurosurgery, King Abdulaziz Medical City, King Saud Bin Abdulaziz University for Health Sciences, Ministry of National Guards Health Affairs, King Abdullah International Medical Research Centre, Jeddah, SAU; 2 College of Medicine, King Abdulaziz Medical City, King Saud Bin Abdulaziz University for Health Sciences, Ministry of National Guards Health Affairs, King Abdullah International Medical Research Centre, Jeddah, SAU; 3 Neurological Surgery, Alnoor Specialist Hospital, Makkah, SAU; 4 Endocrinology, King Faisal Specialist Hospital and Research Center, Jeddah, SAU; 5 Endocrinology, King Abdulaziz Medical City, King Saud Bin Abdulaziz University for Health Sciences, Ministry of National Guards Health Affairs, Jeddah, SAU

**Keywords:** diabetes insipidus, cerebral salt-wasting syndrome, sellar/suprasellar astrocytoma, hydroelectrolytic abnormality post-neurosurgery, post-neurosurgery complication

## Abstract

Water homeostasis disorders, such as syndrome of inappropriate antidiuretic hormone secretion (SIADH), diabetes insipidus (DI), and cerebral salt-wasting syndrome (CSWS), can develop after neurosurgery. Additionally, DI, SIADH, and CSWS have been reported concurrently in association with some neurosurgical conditions, in particular after pituitary gland surgery or as sequelae of post-traumatic brain injury. Therefore, neurosurgeons should expect water homeostasis disorders after the removal of tumors of the sellar/suprasellar region and be prepared to aggressively manage them.

## Introduction

Water homeostasis disorders, such as the syndrome of inappropriate antidiuretic hormone secretion (SIADH), diabetes insipidus (DI), and cerebral salt-wasting syndrome (CSWS), can develop after neurosurgery [1]. The reported incidence of water homeostasis disorders after neurosurgery in children ranged from 15% to 45% [1]. Furthermore, the incidence of the disorders varies, in which transient DI was the most reported disorder, followed by SIADH and CSWS [2,3]. Additionally, DI, SIADH, and CSWS have been reported concurrently after neurosurgery, especially pituitary gland surgeries and post-traumatic brain injury [2,4,5]. In this case report, we present a rare case of a patient with triphasic DI and CSWS, presenting after neurosurgery, and there were difficulties in differentiating the second phase of DI, which resembles SIADH, and CSWS.

## Case presentation

A 16-year-old female presented to the emergency department complaining of progressive vision loss in both eyes for three months associated with headache and intermittent vomiting. On examination, the patient had normal vital signs, and her height and weight were 147 cm and 35.7 kg. The patient was alert and oriented with a Glasgow Coma Scale (GCS) score of 15/15. On examining the pupils, the patient had a right relative afferent pupillary defect. On the visual acuity test, the patient could only perceive light on her right eye and count fingers on her left eye. The fundus of the right eye was mildly pale. Moreover, she had bitemporal hemianopsia and right sixth cranial nerve palsy. However, she had normal muscle strength. Previous brain magnetic resonance imaging (MRI) showed an optic glioma with the involvement of sellar and suprasellar regions, supratentorial ventriculomegaly, and obstructive hydrocephalus.

**Figure 1:**
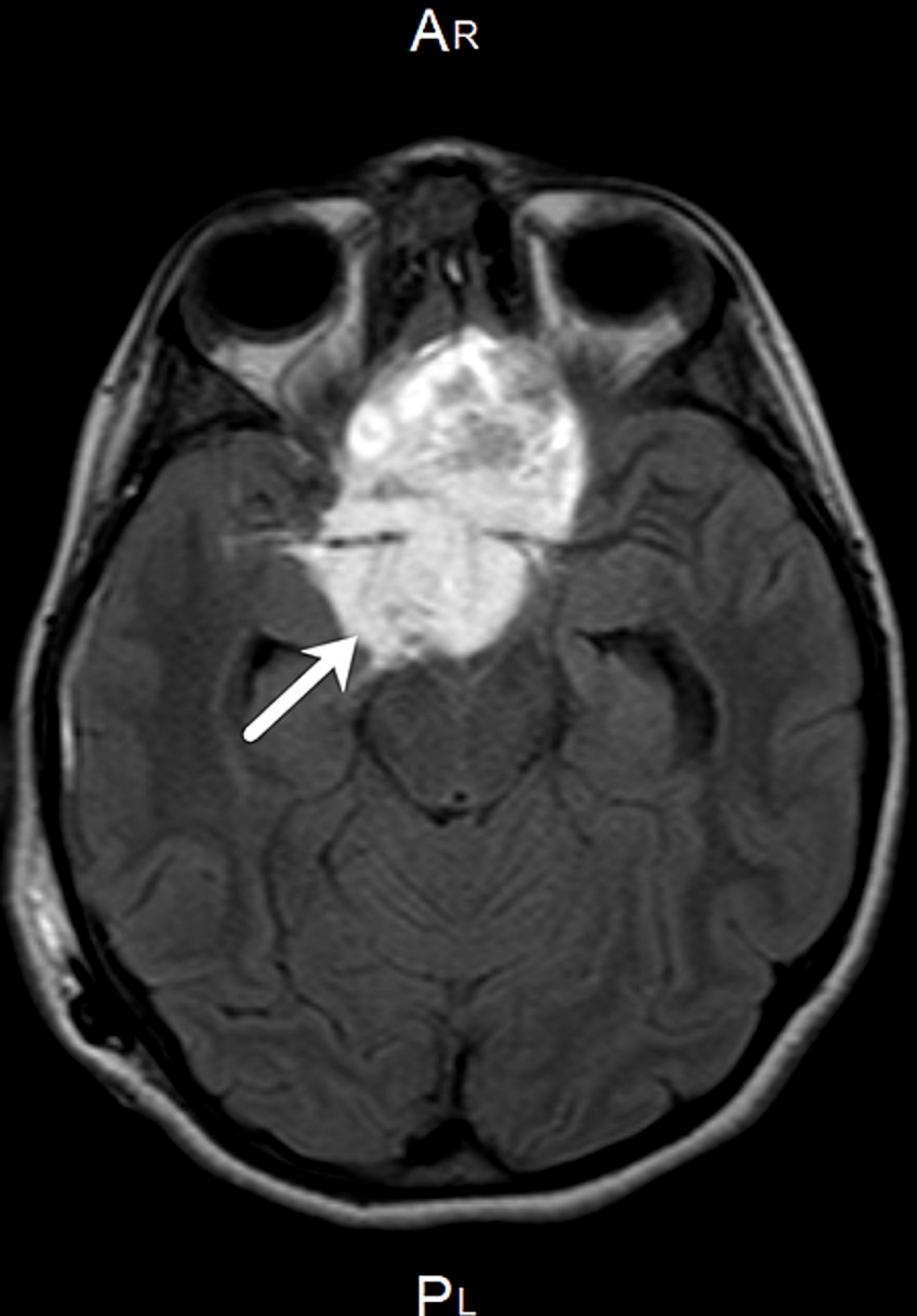
T1-weighted brain MRI post-contrast axial view shows a large sellar/supraseller mass engulfing the anterior aspect of the circle of the Willis and extending to the third ventricle.

**Figure 2:**
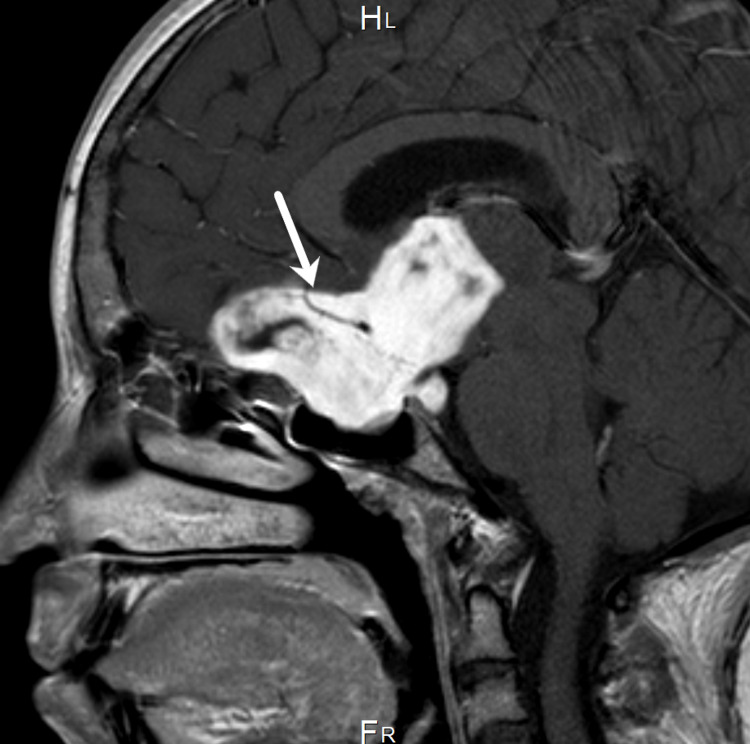
T1-weighted brain MRI post-contrast sagittal view shows the large sellar/supraseller mass engulfing the anterior portion of the circle of the Willis with extension to the third ventricle.

The patient was admitted to the Neurosurgery Department for a right ventriculoperitoneal shunt insertion. On the second postoperative day, the patient was doing well, and baseline pituitary hormones were drawn. The prolactin level (4.52 µg/L) was normal. However, the follicle-stimulating hormone level (0.25 IU/L) and the luteinizing hormone level (<0.5 IU/L) were low. The cortisol (505.0 mcg/dL) and free thyroxine (FT4) (20.39 nmol/L) levels were high, and the adrenocorticotropic hormone (ACTH) (< 1.6 pmol/L) level was low, so the endocrinology team was informed. The increase in cortisol level was due to exogenous steroid intake. A brain MRI was performed on the third day of admission, which showed a large sellar/suprasellar 5.6 × 4.4 × 3.6 cm mass (Figures 1, 2). It was bright on T2-weighted imaging and on T1-weighting and hypointense with heterogeneous enhancement on post-contrast. The lesion was separated from the pituitary gland, which appeared to deviate posteriorly in the sella. A small loculated component extending to the prepontine cistern was observed. Moreover, a large component extending superiorly into the third ventricle was noted. Another large component was found in the olfactory groove. Furthermore, the lesion had engulfed the anterior aspect of the circle of Willis with no mass effect on the vessels. The optic chiasm could not be seen clearly, and bilateral parasellar extension was observed. No edema surrounding the brain parenchyma was found.

On the fifth day of hospitalization, she underwent a partial resection of the tumor using a frontotemporal approach. Both of the optic nerves, olfactory nerves, third cranial nerves, carotid arteries, and anterior cerebral arteries were freed from the tumor. As much of the upper tumor was removed as was safe to resect and thus residual tumor was left. The pathology result showed pilocytic astrocytoma. Postoperatively, the patient was admitted to the Intensive care unit (ICU). After that, the patient developed pupil dilatation and abnormal movements in the form of right arm jerks and muscle rigidity that lasted less than two minutes and aborted spontaneously. The patient received a stat dose of midazolam. After that, the patient was able to wake up and could mobilize. Later, Levetiracetam (Keppra) was started.

Subsequently, the patient had stable vital signs and was kept nothing by mouth (NPO). The patient’s head of the bed was elevated to 30°, and she had postoperative orders written for 500 mg intravenous cefazolin three times a day for two days, 80 mL 20% mannitol three times a day for two days, 50 mg hydrocortisone, one dose postoperatively then 50 mg intravenous hydrocortisone four times a day, pneumatic compression and strict input and output monitoring. On the first postoperative day, the patient’s test results showed an FT4 of 14.30 nmol/L, cortisol of 2,992 nmol/L, ACTH of less than 1.60 pmol/L, creatinine of 37 nmol/L, a blood urea nitrogen of 1.8 mg/dL, and 50 mg intravenous hydrocortisone was continued.

On postoperative day 2 (PO day 2), the patient was discharged from the ICU and complained of polyuria. The patient’s urine output rate reached 499.8 mL/h that started at midnight, and arterial blood gases revealed that her serum sodium (Na) level was 145.6 mEq/L, so mannitol was held. The urine output improved to 4 mL/h, and the patient's serum Na level was 144 mEq/L with normal plasma osmolality (pOsm) (288 mOsm/kg) and urine osmolarity (uOsm), (416 mOsm/kg). In addition, the patient was assessed by the ophthalmology team. The assessment showed that the pupils were mildly dilated with a very sluggish reaction to light, and the visual acuity test showed no light perception in both eyes. Moreover, the patient had diffusely pale optic discs and clear disc margins with a normal macula, vessels, and periphery.

On PO day 3, the patient complained of thirst with a serum Na level of 141 mEq/L, uOsm of 164 mOsm/kg, and pOsm of 291 mOsm/kg. The patient’s condition was interpreted as DI; thus, a stat dose of intravenous desmopressin was administered at 10:30 am, and her intravenous fluid was decreased from 90 mL/h to 75 mL/h. Blood tests were taken after the administration of desmopressin, which showed a serum Na level of 136 mEq/L, pOsm of 283 mOsm/kg, and uOsm of 739 mOsm/kg.

On PO day 4, the patient’s serum Na level started to decrease (124 mEq/L), and the pOsm was 254 mOsm/kg, uOsm was 790 mOsm/kg, and the urine output rate reached 30 mL/h in the last 4 h. The patient was diagnosed with SIADH. The patient was asymptomatic, however. A 2% hypertonic solution at the rate of 31.5 mL/h and 4 mg intravenous dexamethasone every 6 h for seven days were started.

On PO day 5, the patient was readmitted to the ICU due to symptoms of hyponatremia, such as nausea and fatigue (serum Na level, 120 mEq/L). Then, 3% hypertonic saline at a rate of 30 mL/h was started. Additionally, a 25 mcg levothyroxine intravenous push was administered.

On PO day 6, the patient had CSWS with a serum Na level of 118 mEq/L, urine Na level of 264 mEq/L, uOsm of 673 mOsm/kg, and urine specific gravity of 1.015. The rate of hypertonic saline was increased to 50 mL/h. The serum Na then reached 130 mEq/L and the rate of 3% hypertonic saline was decreased to 40 mL/h. The patient received an oral sodium chloride dose of 71 mEq.

On PO day 8, the patient was discharged from the ICU on oral sodium chloride and potassium chloride replacement. The patient was hyponatremic with a serum Na level of 127 mEq/L, potassium level of 3.5 mEq/L, and chlorine level of 100 mEq/L.

On PO day 9, the patient's serum Na level increased from 127 to 132 mEq/L with high urine output; thus, 0.25 mcg desmopressin was administered, and sodium chloride and maintenance intravenous fluid were discontinued. Moreover, 25 mcg oral levothyroxine was initiated due to central hypothyroidism. The patient was kept on salt tablets.

On PO day 13, the Endocrinology Department concluded that the patient had demonstrated a case of grade 1 pilocytic astrocytoma complicated by central hypothyroidism and suspected central adrenal insufficiency, central hypogonadism, and central partial DI or primary polydipsia.

On PO day 16, the patient was discharged and was instructed to follow up with the Neurosurgery, Endocrinology, and Radiation Oncology Departments (Table 1).

**Table 1:** Laboratory results after the surgery

Postoperative day	Normal Laboratory Values	0	1	2	3	4	5	6	7	8	9	10	11	12	13	14
Prolactin (ug/L)	4.2–23.04	-	-	-	-	-	-	-	-	-	-	-	-	-	-	8.50
Cortisol (nmol/L)	76.9–452.5	-	2,992	-	-	-	-	-	-	-	-	-	-	-	-	<28
Thyroid-Stimulating Hormone (TSH) (mIU/L)	0.47–3.41	-	-	-	-	-	-	-	-	-	-	-	-	-	-	0.06
Luteinizing Hormone (LH) (IU/L)	2.39–6.60	-	-	-	-	-	-	-	-	-	-	-	-	-	-	<0.5
Follicle-Stimulating Hormone (FSH) (IU/L)	3.4–21.6	-	-	-	-	-	-	-	-	-	-	-	-	-	-	0.22
Free Thyroxine (FT4) (pmol/L)	10.2–17.3	-	14	-	-	-	10.04	-	-	-	-	-	-	-	-	11
Free Triiodothyronine (FT3) (pmol/L)	3.55–5.7	-	2.49	-	-	-	1.73	-	-	-	-	-	-	-	-	1.72
Adrenocorticotropic Hormone (ACTH) (pg/mL)	4.7–48.8	-	<1.60	-	-	-	-	-	-	-	-	-	-	-	-	<1.60
Random Blood Glucose (mmol/L)	4.4–7.8	-	9.6	8.3	7.1	7.4	4.7	5.5	5.8	5.3	7.9	6.2	7.7	5.8	6.4	5.1
Plasma Na (mEq/L)	135–147	138.7	137.2	145.3	141	124	120	118	127	127	132	135	135	137	133	-
pOsm (mOsm/kg)	288–298	-	-	288	283	254	243	-	-	259	275	280	279	278	-	-
Urine Sodium (mmol/L)	Result varies with dietary intake	44	55	-	-	-	242	264	-	-	-	-	-	-	-	-
uOsm (mOsm/kg)	250–900	729	770	416	153	689	603	673	-	471	108	171	59	48	55	-
Hgb (g/dL)	11.5–16.5	9.0	8.5	7.8	8.5	8.7	8.9	9.8	9.5	10.3	10.3	9.3	-	-	-	-
Hematocrit (%)	40.0–54.0	-	27.1	27	27.6	27.6	-	29.7	-	-	-	-	-	-	-	-
Sodium chloride (NaCl) 2% hypertonic solution (mL/h)	-	-	-	-	-	31.5	-	-	-	-	-	-	-	-	-	-
Mannitol 20% Injection (g)	-	16 (q8h)	16 (q8h)	-	-	-	-	-	-	-	-	-	-	-	-	-
Sodium chloride (NaCl) 3% hypertonic solution (mL/h)	-	-	-	-	-	-	30	30	30	30	-	-	-	-	-	-
Sodium chloride 0.9% (normal saline) (mL/h)	-	-	-	90	500 mL IV bolus X 2 90	60	30	30	-	-	-	-	-	-	-	-
Dextrose 5% in NaCl 0.9 (mL/h)		75	75	75	-	-	50	75 30	75 30	75 30	30	-	-	-	-	-
Sodium chloride oral solution (mL)	-	-	-	-	-	-	-	30	30	30	30	-	-	-	-	-
Potassium chloride injection (meq)	-	-	-	20	20	-	15	15	15	15	10	-	-	-	-	-
Desmopressin (mcg)	-	-	-	-	1	-	-	-	-	-	0.25	-	-	-	-	-
Dexamethasone (mg)	-	-	-	4 (q6h) 5 (q24h)	4 (q6h)	4 (q6h)	4 (q6h)	4 (q6h)	4 (q6h)	4 (q6h)	4 (q6h)	4 (q6h)	-	-	-	-
Hydrocortisone Injection (mg)	-	50 (q6h)	50 (q6h)	50 (q6h)	-	-	-	-	-	-	-	-	-	-	-	-

## Discussion

DI is defined as an inappropriate decrease in antidiuretic hormone (ADH) secretion, which increases urine output to >3L/day with a low osmolarity <300 mOsm/kg [6]. After surgery, DI will rarely present in three phases, which is known as triphasic DI. These three phases start with the early polyuric phase in the first 1-2 days. Second, the antidiuretic phase occurs, during which there will be high secretion of ADH, which usually lasts for 5-8 days. During this phase, the patient presents with hyponatremia and SIADH. The third phase is the final polyuric phase, which can be long-lasting [7]. The SIADH phase is characterized by the release of large amounts of ADH despite the decrease in plasma osmolarity, which decreases water excretion from the kidney, subsequently leading to hyponatremia [8]. In CSWS, patients present with hyponatremia and a decrease in extracellular fluid volume due to sodium loss through the kidney following intracranial disorders such as subarachnoid hemorrhage, head injury, neurosurgery, intracranial neoplasm, and cerebral infection [3,4]. Like this case, patients usually present with DI at the beginning, followed by CSWS [1,2]. This sequence can lead to some difficulties in determining the diagnosis as the second phase of triphasic DI resembles SIADH and CSWS and can show similarities such as hyponatremia and hypouricemia [1]. However, the main difference between these disorders is that hyponatremia in CSWS is associated with a decrease in volume status (hypovolemia), which was disturbed in the patient in this case report [1,2]. Therefore, determining the diagnosis in our patient was difficult.

In children, the incidence of electrolyte abnormalities is 14-45% after neurosurgical operations [1]. After such procedures, especially those cases which involve the hypothalamus or pituitary gland, having any syndrome that can affect hydro-electrolytic balance, such as DI, SIADH, and CSWS, is not rare. However, having a combination of these syndromes in a single patient is rare, as there are only a few cases presented in the literature [2]. In contrast to many studies that showed cases of tumors limited to the sellar/suprasellar region, the tumor in the present case report's patient was invasive and extended to multiple areas [1,2]. Therefore, using the frontotemporal approach was favorable to have more exposure. However, this approach was not reported as a risk factor for the development of DI in contrast to the transsphenoidal approach, which is considered a risk factor for DI [9]. Moreover, the diagnosis in our patient was a grade 1 pilocytic astrocytoma, which was not usually associated with the development of DI, SIADH, and CSWS. In contrast, craniopharyngioma, Rathke’s cleft cysts, and ACTH-secreting pituitary adenomas were reported as risk factors for the development of DI [10,11].

## Conclusions

In conclusion, even though there are some specific pathology and surgical approaches that were reported to be associated with a higher incidence of water homeostasis disorders, neurosurgeons should expect them after surgeries that involve the pituitary and hypothalamus or surgeries that involve the removal of tumors that extend to the sellar/suprasellar region. Additionally, DI and CSWS can occur simultaneously, which can lead to some difficulties in diagnosing patients. Therefore, awareness of these complications may facilitate the management of the patient. Also, further research to study the risk factors of water hemostasis disorders is required to improve the quality of patient care.
